# Detection of selected bacterial species in intraoral sites of patients with chronic periodontitis using multiplex polymerase chain reaction

**DOI:** 10.1590/S1678-77572010000400018

**Published:** 2010

**Authors:** Cyntia Rodrigues de Araújo ESTRELA, Fabiana Cristina PIMENTA, Ana Helena Gonçalves de ALENCAR, Luis Fernando Naldi RUIZ, Carlos ESTRELA

**Affiliations:** 1 DDS, MSc, PhD, Institute of Biological Sciences, Federal University of Goiás, Goiânia, GO, Brazil.; 2 MSc, PhD, Professor of Microbiology, Institute of Tropical Pathology and Public Health, Federal University of Goiás, Goiânia, GO, Brazil.; 3 DDS, MSc, PhD, Professor of Endodontics, Department of Oral Science, Federal University of Goiás, Goiânia, GO, Brazil.; 4 DDS, MSc, PhD, Professor of Periodontics, Department of Oral Science, Federal University of Goiás, Goiânia, GO, Brazil.; 5 DDS, MSc, PhD, Chairman and Professor of Endodontics, Department of Oral Science, Federal University of Goiás, Goiânia, GO, Brazil.

**Keywords:** Chronic periodontitis, Bacteria, DNA probes, PCR, epidemiological studies

## Abstract

**Objective:**

The aim of this study was to detect the prevalence of selected bacterial species in intraoral sites of patients with chronic periodontitis (CP) using multiplex polymerase chain reaction (PCR).

**Methodology:**

Samples were collected from the tongue dorsum, buccal mucosa, supragingival and subgingival plaque and saliva of 30 patients with untreated CP. Multiplex PCR was used to determine prevalence rates, which were then compared using a chi-square test. Significance level was set at p<0.05. Mean and standard deviation values were used to evaluate variations in prevalence according to site.

**Results:**

The prevalence of *S. mutans* was 70% in saliva; 60% in samples collected from the tongue dorsum; 50% in samples collected from the buccal mucosa; 56.5% in the supragingival plaque; and 53.5% in the subgingival plaque. The prevalence of *E. faecalis* ranged from 3.5% to 13.5% in all intraoral microenvironment. The highest prevalence of *P. gingivalis* was found in subgingival plaque (53.5%), and of *P. intermedia* in supragingival plaque (33.5%), subgingival plaque (30%) and tongue dorsum (33.5%). The prevalence of bacteria did not vary significantly among the intraoral sites.

**Conclusions:**

All studied bacteria were identified in intraoral sites. *S. mutans, P. gingivalis* and *P. intermedia* had high prevalence rates, but the prevalence of *E. faecalis* was low. Multiplex PCR proved to be an adequate method for epidemiological studies.

## INTRODUCTION

More than 700 species of microorganisms, such as Gram-positive and Gram-negative bacteria, yeast, protozoa and viruses, are found in the oral cavity^21^. Microorganisms are distributed in four oral ecosystems - tongue dorsum, buccal mucosa, supragingival and subgingival plaques. Saliva keeps direct contact with oral tissues; it contains cells from different sites of the mouth that may spread as plaque or planktonic suspension^7^.

*Streptococcus mutans, Enterococcus faecalis, Porphyromonas gingivalis, Prevotella intermedia* are bacterial species that knowingly cause several oral diseases, such as dental caries, endodontic infections and periodontal diseases^7,14,19,27^.

*S. mutans* are Gram-positive facultative bacteria that have been implicated as a primary cause of dental caries in humans. One of the important virulence properties of these microorganisms is the ability to form a biofilm, known as dental plaque, on tooth surfaces^13,14^. Some of the bacterial components associated with the adhesion phase of *S. mutans* are glucosyltransferases, protein antigen C and glucan-binding proteins. The number and distribution of genotypes of *S. mutans* isolated from caries-active and caries-free children were used to evaluate some of their phenotypic traits. There were differences in the distribution of genotypes of *S. mutans according to the oral site, and S. mutans* populations differ in their susceptibility to acid and ability to form plaque, factors that lead to their colonization of sucrose-rich environments^13^.

*E. faecalis* has an important role in endodontic infections because of particular strategies to form plaque, substantial virulence factors, adherence to dentin collagen, survival in critical environments, and resistance to endodontic therapy. *E. faecalis* is a Gram-positive coccus, a facultative anaerobe, found in normal human gastrointestinal infections and common in secondary apical periodontitis. Enterococci are classified as the second to third most common organisms found in hospital-acquired infections, and 85-95% of these isolates are *E. faecalis*^19^.

*P. gingivalis* is a Gram-negative anaerobe that resides in subgingival plaque and is associated with severe and chronic cases of periodontal disease, a condition characterized by destruction of the tissue supporting the teeth^4^. A number of factors are associated with the virulence of this oral anaerobe, including a variety of proteases, endotoxins, and collagenase, as well as the production of surface structures such as fimbriae and capsular polysaccharides^10^.

*P. intermedia* are also Gram-negative anaerobes found in the orange complex^25^; they are blackpigmented Bacteroides prevalent in acute necrotizing ulcerative gingivitis^15^ due to CP progression^7^. This species has several of the virulent properties of *P. gingivalis* and induces mixed infections when inoculated in laboratory animals^9^. Its virulence factor is determined by its capability of adhesion to hard surfaces and soft tissues of the oral cavity^6^.

Several studies used polymerase chain reaction (PCR) to detect selected bacterial species in the oral cavity^7,12,20,22^. The multiplex variant of PCR can be used for the simultaneous amplification of two or more loci in the same reaction^23^.

Success in the treatment of intraoral infectious diseases depends on the knowledge of the prevalence of the bacterial species associated with etiological factors. Therefore, this study detected *S. mutans, E. faecalis, P. gingivalis* and* P. intermedia* in several intraoral sites of patients with chronic periodontitis using multiplex PCR technique.

## MATERIALS AND METHODS

### Subjects

Thirty adults with chronic periodontitis (22 women; mean age: 37±5 years) and teeth with vital pulps were selected from a group of patients seeking treatment at the Brazilian Dental Association in Goiânia, GO, Brazil. All participants were in good general health.

Patients were included in the study if they had a clinical diagnosis of chronic periodontitis and no history of odontogenic pain. exclusion criteria were: pregnancy, systemic problems that might affect periodontal disease activity; or use of antimicrobial medication or antimicrobial treatment in the 3 months before inclusion in the study. None of the patients included in the study had previously received any type of periodontal treatment. All patients signed an informed consent form before inclusion, and the study protocol was approved by the Research ethics Committee of the Institute of Biological Sciences of Goiás Federal University, Goiânia, GO, Brazil (protocol # 65/2006, ICB, UFG).

### Clinical examination

Clinical measurements were performed at six intraoral sites (mesiobuccal, buccal, distobuccal, distolingual, lingual and mesiolingual positions) on each tooth except the third molar at a baseline visit. Clinical parameters measured were: pocket depth (mm); bleeding on probing (0 or 1); suppuration (0 or 1); and plaque accumulation (0 or 1). Saliva, oral mucosa and supra- and subgingival plaque samples for microbiological assessment were collected before clinical measurements. All patients provided samples of soft tissue and supra- and subgingival plaque. This model of clinical examination was described in previous studies^17,26^.

### Sampling procedures

Samples were collected from saliva, buccal mucosa, tongue dorsum and supra- and subgingival plaques, in this sequence. Aseptic techniques were used during all the study.

Saliva samples were collected by immersing two sterilized #50 absorbent paper points (Tanari, Tanariman Indústria, Ltda., Manacaru, AM, Brazil) in non-stimulated saliva for 1 minute; the paper points were then placed in eppendorf-like tubes containing 500 µL of sterile PBS buffer. For the other intraoral sites, samples were collected by gently rubbing epithelial and dental surfaces for 1 minute with two #50 sterile absorbent paper points. Supraand subgingival plaque samples were collected from 2 different sites. Samples from supragingival plaque were collected with gently rubbing dental surfaces with two paper points and samples from subgingival plaque were collected with a two sterile #50 absorbent paper points introduced into the periodontal pocket maintained for 1 min. The absorbent paper points were placed in eppendorflike tubes containing 500 µL of sterilized PBS buffer. All the samples were immediately frozen at -20°C for posterior analysis.

The teeth selected for the collection of supra- and subgingival plaque samples had no caries and were vital; pulp vitality was tested with dichlorodifluoromethane spray (cold test, -20°C, Aeroget, Ind. Bras., São Paulo, SP, Brazil).

### Bacterial strains and DNA isolation

Samples were evaluated using multiplex PCR. For DNA extraction, the samples were thawed under refrigeration, vortex-mixed for 1 min, and then centrifuged at 5000 rpm for 5 min. The initial volume of 500 µL was reduced to 300 µL to increase the concentration of microbial cells. Samples were boiled in water bath for 10 min, and then frozen at -20ºC. Reference DNA for the microorganisms under analysis (*S. mutans –* ATCC 25175*, E. faecalis -* ATCC 29212*, P. gingivalis -* ATCC 33277 and *P. intermedia –* ATCC 25611) were also extracted to serve as positive controls.

### PCR assay

The PCR reaction to determine the presence of all microorganisms was performed in 25 µl of reaction mixture containing 13.05 µL of ultrapure water, 2.5 µL of 10X PCR Buffer (Invitrogen, Carlsbad, CA, USA), 1.75 µL of 50 mM MgCl2 (Invitrogen, Carlsbad, CA, USA), 1.0 µL of each primer (50 mM/ µL) (*E. faecalis* F – GTT TAT GCC GCA TGG CAT AAG AG, *E. faecalis* R – CCG TCA GGG GAC GTT CAG, *S. mutans* F – ACT ACA CTT TCG GGT GGC TTG G, *S. mutans* R – CAG TAT AAG CGC CAG TTT CAT C) (Invitrogen, Carlsbad, CA, USA), 0.5 µL of 10 mM dNTP Mix (ie, deoxyadenosine triphosphate, deoxycytidine triphosphate, deoxyguanosine triphosphate, and deoxythymidine triphosphate; Invitrogen, Carlsbad, CA, USA) and 0.2 µL of Taq DNA Polymerase (Invitrogen, Carlsbad, CA, USA). Ultrapure water was used in the place of bacterial DNA template in each PCR reaction as a negative control, and extracted DNA from the reference microorganisms (ATCC) was used as a positive control.

PCR amplification was performed in a DNA thermocycler (Mastercycler Gradient, eppendorf, Hamburg, Germany). Thermal cycling parameters for *E. faecalis* and *S. mutans* were: initial denaturation at 95ºC for 5 min, followed by 36 cycles of denaturation at 95ºC for 30 s, primer-annealing at 60ºC for 1 min, extension at 72ºC for 1 min, and a final step at 72ºC for 10 min; for *P. gingivalis* and *P.* *intermedia*, initial denaturation at 94ºC for 5 min, followed by 36 cycles of denaturation at 94ºC for 1 min, primer-annealing at 56ºC for 1 min, extension at 72ºC for 2 min, and a final step at 72ºC for 10 min. (*P. gingivalis* F - AGG CAG CTT GCC ATA CTG CG, *P. gingivalis * R - ACT GTT AGC AAC TAC CGA TGT, *P. intermedia* F – CGT GGA CCA AAG ATT CAT CGG TGG A, *P. intermedia* R – CCG CTT TAC TCC CCA ACA AA).

The PCR amplicons were analyzed using electrophoresis in 1% agarose gel (Invitrogen, Carlsbad, CA, USA) in Tris-borate-eDTA buffer. The gel was stained for 30 min with 1 mg/ml ethidium bromide and visualized under ultraviolet light. Reactions were classified as positive when bands of expected sizes were detected. A Lambda Hind (Invitrogen, Carlsbad, CA, USA) 100 bp ladder was used as a size marker.

The prevalence of bacteria in intraoral sites was analyzed with a chi-square test, and the level of significance was set at p<00.5 (SPSS; Inc., v.15 for Windows, Chicago, IL, USA). Mean and standard deviation values were used to evaluate the variations of bacterial prevalence rates according to intraoral sites.

## RESULTS

The bacteria selected and their prevalence in intraoral sites are shown in Table 1. The depth of periodontal pockets in 55% of the cases was ≥ 4mm, and in 45%, ≥6 mm. *S. mutans* was detected in 70% of saliva samples; 60% of tongue dorsum samples; 50% of buccal mucosa samples; 56.5% of supragingival plaque samples; and 53.5% of subgingival plaque samples.

**Table 1:** Prevalence of bacterial species in samples from different intraoral sites

**Bacteria**	**Intraoral sites**	**n**	**Presence**	**X^2^**	**p**
					
S.*mutans*	Saliva	30	21 (70%)	2.901	0.575
	Tongue dorsum	30	18 (60%)		
	Buccal mucosa	30	15 (50%)		
	Supragingival plaque	30	17 (56.5%)		
	Subgingival plaque	30	16 (53.5%)		
*E. faecalis*	Saliva	30	1 (3.5%)	3.214	0.523
	Tongue dorsum	30	4 (13.5%)		
	Buccal mucosa	30	1 (3.5%)		
	Supragingival plaque	30	2 (6.5%)		
	Subgingival plaque	30	2 (6.5%)		
*P. gingivalis*	Saliva	30	7 (23.5%)	8.734	0.068
	Tongue dorsum	30	7 (23.5%)		
	Buccal mucosa	30	9 (30%)		
	Supragingival plaque	30	12 (40%)		
	Subgingival plaque	30	16 (53.5%)		
*P. intermedia*	Saliva	30	7 (23.5%)	1.093	0.895
	Tongue dorsum	30	10 (33.5%)		
	Buccal mucosa	30	8 (26.5%)		
	Supragingival plaque	30	10 (33.5%)		
	Subgingival plaque	30	9 (30%)		

**Figure 1:**
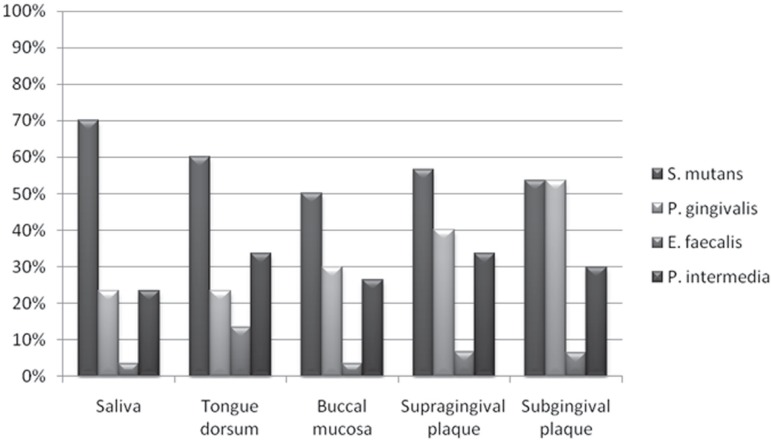
Frequency of selected bacterial species in different intraoral sites

The prevalence of *E. faecalis* was 3.5% in saliva; 13.5% in the tongue dorsum; 3.5% in the buccal mucosa; 6.66% in supragingival plaque; and 6.5% in subgingival plaque. The prevalence of *P. gingivalis* was 23.5% in both saliva and the tongue dorsum; 30% in the buccal mucosa; 40% in supragingival plaque; and 53.5% in subgingival plaque. For *P. intermedia*, prevalence rates were 23.5% in saliva samples; 33.5% in the tongue dorsum; 26.5% in the buccal mucosa; 33.5% in supragingival plaque; and 30% in subgingival plaque (Figure 1). There were no statistically significant differences (p>0.05) between saliva and samples collected from the other intraoral sites for the bacteria studied. Bacterial prevalence remained independent from site analyzed, which was demonstrated by the relatively constant value of standard deviations for the studied sites (according to type of bacteria). Mean prevalence values for *P. intermedia* were the closest to the general mean prevalence rates according to sites investigated.

## DISCUSSION

Oral diseases, such as dental caries and endodontic and periodontal infections, are often associated with important etiological agents, such as *S. mutans, E. faecalis, P. gingivalis* and* P. intermedia*.

The determination of bacterial prevalence rates in all sites under study at the same time and in association with CP was an important characteristic of this study because saliva establishes direct contact between four oral ecosystems, which are important reservoirs for infection.

The bacteria selected for this study play an important role in oral microbial ecology. The microorganisms that colonize the oral environment produce plaques of different complexities depending on intraoral site, genetic background and individual environmental factors^7^. Socransky and Haffajee^25^ (1991) reported that once periodontal tissues are colonized, evidence suggests that only a subsequent species causes destructive periodontal disease. To colonize subgingival sites, bacteria must attach to one or more of the available surfaces, multiply, compete successfully against other species for that habitat, and defend itself from host defense mechanisms.

The prevalence of *S. mutans* in the studied microenvironments was elevated (greater than 50%). This bacterial species is the one most often studied in the human mounth^14,20^. Wu, et al.^29^ (2003) found that the prevalence of *S. mutans* in supragingival plaques in individuals with dental caries was 75.4%. The prevalence rate in our study was lower (56.5%), but participants’ teeth were free of caries.

The prevalence of *E. faecalis* was lower in all intraoral sites (3.5% to 13.5%). This type of bacteria is common in gastrointestinal infections and secondary endodontic infections. Colombo, et al.^2^ (2002) determined the subgingival microbiota of 25 patients with untreated CP using the Checkerboard DNA-DNA hybridization technique and found some unusual types of *E. faecalis* . Slots, et al.^24^ (1991) identified a prevalence of 23% of enteric bacteria in patients with periodontitis.

In the present study, teeth from which samples of sub- and supragingival plaques were collected showed pulp vitality and absence of dental caries.

*P. gingivalis* was found in 23.5% of saliva and tongue dorsum samples, 30% of buccal mucosa samples, 40% of supragingival plaque samples, and 53.5% of subgingival plaque samples. There were no significant statistical differences between the sites analyzed (p=0.068). These results are in agreement with previous findings. Wahlforst, et al.^28^ (1995) studied subgingival plaques of 36 patients with CP and found a prevalence of *P. gingivalis* of 56% using PCR and 42% using cultures. Mombelli, et al.^18^ (2001) found a prevalence rate of 59% in subgingival plaques after treatment and advanced periodontal disease. Yano-Higuchi, et al.^31^ (2000) found a prevalence of 64.3% of *P. gingivalis* in subgingival plaques of 21 subjects with adult periodontitis using cultures. Kumar, et al.^11^ (2003) examined subgingival plaques of 66 patients with CP, and found a prevalence of 88% of *P. gingivalis* using PCR. Gajardo, et al.^5^ (2005) detected a prevalence of 76.4% of *P. gingivalis* in subgingival plaques of patients with CP using cultures. Cortelli, et al.^3^ (2005) found a prevalence of 76% of *P. gingivalis* in subgingival plaques of 203 subjects with CP using PCR. Ledder, et al.^12^ (2007) found a prevalence of 29% of *P. gingivalis* in subgingival plaques of 47 subjects with CP using PCR multiplex.

*P. intermedia* had a prevalence rate of 23.5% in saliva, 33.5% in tongue dorsum, 26.5% in buccal mucosa, 33.5% in supragingival plaque and 30% in subgingival plaque. There were no significant statistical differences between the sites analyzed (p=0.895). According to López, et al.^16^ (2000), the prevalence of *P. intermedia* in subgingival plaques of 60 subjects with CP was 33% in a study using DNA probes. Mombelli, et al.^18^ (2001) found a prevalence of 40.6% in subgingival plaques after treatment of advanced periodontitis using cultures. Gajardo, et al.^5^(2005) found a prevalence of 35.2% of *P. intermedia* in subgingival plaques of patients with CP using cultures.

Ximenez-Fyvie, et al.^30^ (2000) compared the microbial composition of supra- and subgingival plaques in 23 patients with adult periodontitis. Periodontal pathogens were detected in supragingival plaque from sites in which subgingival samples were negative for the same species. Supragingival plaque can harbor putative periodontal pathogens, which suggests that this environment may be a reservoir of such species and for the spread of infection or reinfection of subgingival sites.

Mager, et al.^17^ (2003) analyzed the proportions of bacterial species in samples from oral soft tissue surfaces and saliva in healthy adults and compared microbiotas with those of supra- and subgingival plaque. In average, all of the species tested were found in all of the sampled surfaces. The major differences were in the proportion of colonization of the different surfaces, which suggests that receptors, co-aggregation or local habitat differences may play major roles in defining community structure. The microbial composition of saliva was most similar to that found on the lateral and dorsal surfaces of the tongue, suggesting that these surfaces may be the major sources of salivary bacteria. The microbiotas colonizing the remaining surfaces showed great similarities to each other, but differences were detected between surface sites. Plaques on teeth were somewhat similar to each other, but quite different from the microbiotas on soft tissue surfaces and in saliva. However, as pointed out above, tooth-colonizing species were detected on soft tissues. Haffajee, et al.^8^ (2008) observed that in supragengival biofilm samples were similar to those found in subgingival plaque samples with a few minor differences.

The results of the present study are in agreement with values found in other investigations, particularly when some characteristics of variables and methods are taken into consideration^2,11,12,14,19,20,23,27,28,30^.

The method used in this study confirmed results obtained by other authors^1,20^, which indicated that the PCR method can be used in epidemiological studies to analyze the prevalence of selected bacteria in different populations. According to Oho, et al.^20^ (2000), PCR is simpler than the traditional culture methods and provides results in a few hours. Colombo, et al.^1^ (1998) reported that the levels of ‘‘classic’’ periodontal pathogens such as *B. forsythus, P. gingivalis* and *A. actinomycetemcomitans* do not appear to be elevated in most refractory patients. According to their study, these organisms should be detected by more sensitive means, such as PCR, to examine their roles in periodontal diseases.

The risks and benefits of molecular techniques to achieve the main objective of this study indicate that multiplex PCR is adequate to determine the prevalence of bacteria in several intraoral sites.

## CONCLUSIONS

All the bacterial species under study were identified in intraoral sites of our patients. The prevalence rates for *S. mutans, P. gingivalis* and *P. intermedia* were high in intraoral sites, but low for *E. faecalis*. Multiplex PCR proved to be an adequate method for epidemiological studies.
